# Meteorological Report for September, 1879

**Published:** 1879-11

**Authors:** H. Hall

**Affiliations:** Corp’l. Signal Service U.S. A.; Atlanta, Ga.


### METEOROLOGICAL REPORT FOR SEPTEMBER, 1879.

| RAIN. DATE | IN. | Mean daily Barometer. | Mean daily Thermometer | Mean daily Humidity. | Maximum Thermometer | Minimum Thermometer | Prevailing direction of Wind. | Maximum hourly velocity of Wind, (Daily.) |
|---|---|---|---|---|---|---|---|---|
| 1 | 23 | 29.793 | 74 7 | 82.7 | 79 | 71 | E. | 19 |
| 2 | 1.04 | 29.717 | 79.2 | 81.7 | 81 | 72 | S. E. | 28 |
| 3 | 0 | 29 891 | 74.7 | 65.7 | 80 | 65 | N. W. | 13 |
| 4 | 0 | 29.995 | 71.7 | 66.3 | 78 | 65 | N. W. | 15 |
| 5 | 0 | 30.041 | 73.5 | 65.7 | 81 | 64 | E. | 9 |
| 6 | 0 | 30.068 | 74 7 | 58 0 | 82 | 66 | N. | 8 |
| 7 | 0 | 30.029 | 79.2 | 56.7 | 86 | 66 | S. W. | 9 |
| 8 | 0 | 29 998 | 74.7 | 63.0 | 84 | 70 | N. W. | 15 |
| 9 | 0 | 30 091 | 67.0 | 57.3 | 74 | 57 | N. | 12 |
| 10 | 0 | 30 188 | 67.2 | 57.0 | 76 | 57 | N. E. | 13 |
| 11 | 0 | 30.239 | 67 0 | 66.3 | 74 | 60 | E. | 14 |
| 12 | 0 | 30.124 | 72 0 | 53.0 | 77 | 60 | S. E. | 12 |
| 13 | 13 | 30.066 | 64.2 | 81.3 | 71 | 63 | S. E | 13 |
| 14 | 01 | 30 023 | 65.2 | 62.3 | 73 | 60 | N. W. | 11 |
| 15 | 0 | 30 025 | 66.7 | 63.3 | 76 | 56 | E | 15 |
| 16 | 0 | 29.990 | 70.2 | 57.3 | 78 | 60 | N. W. | 12 |
| 17 | 0 | 30 063 | 72.5 | 51 3 | 82 | 61 | N. W. | 13 |
| 18 | 0 | 30 073 | 73 7 | 46.3 | 83 | 61 | N. E. | 14 |
| 19 | 0 | 30.053 | 73.7 | 56.3 | 83 | 64 | N. E. | 16 |
| 20 | 0 | 30 023 | 69.0 | 70.7 | 74 | 63 | N. E. | 18 |
| 21 | 0 | 30 028 | 65 5 | 77.3 | 71 | 63 | N. E. | 22 |
| 22 | 0 | 30.061 | 64.7 | 76.3 | 69 | 60 | N. E. | 21 |
| 23 | 0 | 30.129 | 65.2 | 80.3 | 71 | 62 | NE&SE | 13 |
| 24 | ...... | 30.093 | 69.5 | 73.3 | 77 | 63 | N. W. | 14 |
| 25 | 0 | 30.137 | 64.0 | 61.0 | 72 | 55 | N. W. | 16 |
| 26 | 0 | 30.245 | 59.0 | 53.7 | 68 | 51 | E. | 19 |
| 27 | 0 | 30 248 | 60.5 | 61.0 | 71 | 44 | E. | 15 |
| 28 | 0 | 30.211 | 63.2 | 51.3 | 75 | 51 | E. | 13 |
| 29 | 0 | 30 190 | 68.5 | 57.7 | 78 | 54 | E. | 19 |
| 30 | 02 | 30.271 | 68.2 | 89.7 | 72 | 62 | E. | 16 |
| 31 | ...... | ......... | ...... | ...... | ... | ... | ......... | ......... |
| Monthly Means. | | 30 070 | 69.3 | 64.8 | 76.5 | 60.9 | | |

Highest Barometer, 30.336 on the 27th, at 7 and 7 o'clock, A.M.
Lowest Barometer, 29.615, on the 2nd, at 4 o'clock, P.M.
Monthly range of Barometer, 0.721.
Highest Temp. 86°, on 7th, Lowest Temp. 44°, on 27th.
Monthly range of Temperature, 42°.
Greatest daily range of Temperature, 27°, on the 27th.
Lowest daily range of Temperature, 18°, on the 14th, 13th and 21st.
Mean of Maximum Temperatures, 76 5.
Mean of Minimum Temperatures, 60.9.
Mean daily range of Temperature, 15.6. Total rainfall, 1.43 inches.
Prevailing wind, East. Total movement of wind, 64.88 miles.
Maximum velocity of wind and direction, 28 miles per hour, S. E., 2nd.
Number Cloudy days on which rain fell, 6.
Number of cloudy days on which no rain fell, 4.
Total number of days on which rain fell, 6.

H. HALL, Corp'l. Signal Service U. S. A.

Atlanta, Ga., Sept. 1, 1879.

